# Understanding ownership of patient care: A dual-site qualitative study of faculty and residents from medicine and psychiatry

**DOI:** 10.1007/s40037-017-0389-2

**Published:** 2017-12-05

**Authors:** Deborah S. Cowley, Jesse D. Markman, Jennifer A. Best, Erica L. Greenberg, Michael J. Grodesky, Suzanne B. Murray, Kelli A. Corning, Mitchell R. Levy, William E. Greenberg

**Affiliations:** 1 Department of Psychiatry and Behavioural Sciences, University of Washington, Seattle, WA USA; 2 Department of Medicine, University of Washington, Seattle, WA USA; 3 Department of Psychiatry, Harvard University, and Massachusetts General Hospital, Boston, MA USA; 4 Department of Psychosocial and Community Health, University of Washington, Seattle, WA USA; 5 Department of Psychiatry, Harvard University and Beth Israel Deaconess Medical Center, Boston, MA USA

**Keywords:** Ownership, Professionalism, Resident education

## Abstract

**Introduction:**

With changes in duty hours and supervision requirements, educators have raised concerns about erosion of patient care ownership by resident physicians. However, the definition of ownership is unclear. This qualitative study investigated definitions of ownership in medicine and psychiatry faculty and residents.

**Methods:**

The authors distributed an anonymous online survey regarding definitions of ownership to faculty and residents at the psychiatry and internal medicine residency programs at the University of Washington and the Harvard Longwood psychiatry residency and conducted a qualitative analysis of free-text responses to identify emergent themes.

**Results:**

225 faculty (48.6%) and 131 residents (43.8%) across the three programs responded. Responses yielded themes in five domains: Physician Actions, Physician Attitudes, Physician Identity, Physician Qualities, and Quality of Patient Care. All groups identified themes of advocacy, communication and care coordination, decision-making, follow through, knowledge, leadership, attitudes of going ‘above and beyond’ and ‘the buck stops here’, responsibility, serving as primary provider, demonstrating initiative, and providing the best care as central to ownership. Residents and faculty had differing perspectives on ‘shift work’ and transitions of care and on resident decision-making as elements of ownership.

**Discussion:**

This study expanded and enriched the definition of patient care ownership. There were more similarities than differences across groups, a reassuring finding for those concerned about a decreasing understanding of ownership in trainees. Findings regarding shared values, shift work, and the decision-making role can inform educators in setting clear expectations and fostering ownership despite changing educational and care models.

## What this paper adds

In recent years, educators have expressed concern about erosion of ‘ownership’ among resident physicians. However, the definition of ownership is inconsistent and lacks systematic study. This qualitative study of residents and faculty in medicine and psychiatry at two institutions provides a rich understanding of meanings and components of ownership. The robust set of core elements identified across all groups in this study, as well as the differences between faculty and residents in perspectives on ‘shift work’ and the importance of resident decision-making, provide a framework for an enhanced conceptual understanding of and educational interventions to promote ownership of patient care.

## Introduction

With changes in duty hours and supervision requirements, educators have expressed concerns about erosion in residents’ ‘ownership’ of patient care [1–6]. Though the term ownership is used in discussions of professionalism and medical education, systematic studies of the definition of this term are lacking and existing references lack consistency. For example, ownership has been described as ‘knowing everything about one’s patients and doing everything for them’ [3]; ‘the feeling of accountability’ for a patient and a care plan [4]; being assigned the care of a patient 24 h a day, 7 days a week; being responsible for the patient’s management and eventual disposition; and being the one person in charge of decision-making [6–8]. Ownership has also been conceptualized as resulting from time invested in the patient’s care and in a longitudinal treatment relationship [6–8] and has been linked with professional attributes of commitment (‘being obligated or emotionally impelled to act in the best interest of the patient’) and presence (‘to be fully present for a patient without distraction and to fully support and accompany the patient throughout care’) [9, 10].

Given the increasing use of the term ownership, it is important to better understand what meanings are ascribed to this term by faculty and residents to provide a foundation for efforts to promote ownership in graduate medical education. In a pilot study within the Psychiatry residency program at one of our institutions, faculty and residents were asked to define ownership of patient care [11]. Both groups agreed that ownership included essential elements of advocacy, autonomy, commitment, communication, follow-through, knowledge, and teamwork. Faculty, but not residents, identified additional elements of continuity of care, excellence, spending extra time, initiative, and a sense of vocation or calling. Themes identified only by residents were hierarchical tension (i. e. struggles with faculty regarding autonomy and independence) and leadership or being in charge of the patient’s care.

The objectives of the current qualitative study were to: 1) broaden the exploration of the definition of ownership by studying residents and faculty in two different Psychiatry programs and an Internal Medicine program; 2) identify specific behaviours felt by internists and psychiatrists to exemplify ownership; and 3) determine whether ownership definitions or behaviours vary between these two specialties and between residents and faculty. This was a qualitative study that aimed to elicit as broad as possible a collection of meanings and experiences related to ownership and thereby identify components of this concept.

## Methods

### Settings and sample

This study was conducted at the University of Washington, Seattle, Washington, and Beth Israel Deaconess Medical Center, Boston, Massachusetts. We utilized stratified purposeful sampling of residents and faculty from three programs: the University of Washington Psychiatry Residency (UW Psychiatry), Harvard Longwood Psychiatry Residency (HL Psychiatry), and University of Washington Internal Medicine Residency (UW Medicine).

This study was granted exempt status by the University of Washington Human Subjects Division for both the Department of Medicine survey (4/18/2012; HSD study #42732) and the Department of Psychiatry and Behavioural Sciences survey (5/10/2012; HSD study #42964) and by the Beth Israel Deaconess Medical Center Committee on Clinical Investigations (3/30/2012; protocol #2012P-000112).

### Data collection

Qualitative narrative responses were obtained between April and August 2012 using an anonymous electronic communication. We asked two structured, open-ended questions, as follows:

1. Please define ownership of patient care.
2. Please provide one or two examples of behaviours that indicate that a physician is taking ownership of patient care.

We also asked multiple choice questions regarding age and postgraduate year or years post-training.

Non-respondents received two electronic reminders within the 3 weeks after initial survey distribution. This study was reviewed by the Institutional Review Boards of the University of Washington and Beth Israel Deaconess Medical Center and was determined by both bodies to be exempt.

### Research design

We utilized a qualitative, descriptive design to elicit in-depth responses from those sampled.

### Data analysis

The research teams comprised members of each program and each group identified a team of coders. Since the UW Psychiatry group had conducted a prior pilot study [11], we included some coders who had not participated in the coding from that study. The coders employed bracketing, that is, consciously refraining from referring to prior coding themes, to minimize the introduction of *a priori* beliefs, attitudes and perceptions.

Each team conducted open coding of faculty and then resident responses. Following open coding, each group engaged in axial coding utilizing the constant comparative method [12] to refine, synthesize and saturate the categories. Each team developed their section of a codebook (one each for residents and faculty) citing the emerging codes and data-grounded examples of their meanings. After the groups completed this process separately, there were multiple sessions of code refinement and synthesis with the three combined teams.

## Results

### Response rates and sample characteristics

Survey response rates were similar across the three programs: 49.6% (116/234) for faculty and 40.5% (68/168) for residents from UW Medicine, 48.5% (33/68) for faculty and 50.0% (37/74) for residents from UW Psychiatry, and 47.2% (76/161) for faculty and 45.6% (26/57) for residents from HL Psychiatry.

Data regarding age, postgraduate year of residents, and number of years post-training of faculty are shown in Table 1. Most residents were below, and most faculty members above, the age of 35. Residents from all postgraduate years were represented. Most faculty members were 6 or more years post training.

**Table 1:** Resident and faculty ages and postgraduate year or years post-training

	UW Psychiatry^a^	HL Psychiatry^a^	UW Medicine^a^
*Residents*			
Age (years)			
<35	26 (70.3%)	25 (96.2%)	62 (91.2%)
35–44	4 (10.8%)	1 (3.8%)	4 (5.9%)
45 or older	2 (5.4%)	0 (0.0%)	0 (0.0%)
No response	5 (13.5%)	0 (0.0%)	2 (2.9%)
Residency year			
PGY-1	5 (13.5%)	10 (38.5%)	19 (27.9%)
PGY-2	9 (24.3%)	7 (26.9%)	22 (32.4%)
PGY-3	12 (32.4%)	5 (19.2%)	25 (36.8%)
PGY-4	10 (27.0%)	4 (15.4%)	2 (2.9%)
No response	1 (2.7%)	0 (0.0%)	0 (0.0%)
*Faculty*			
Age (years)			
<35	5 (15.2%)	6 (7.9%)	7 (6.0%)
35–44	12 (36.4%)	12 (15.8%)	53 (45.7%)
45–54	8 (24.2%)	14 (18.4%)	29 (25.0%)
55 or older	8 (24.2%)	44 (57.9%)	27 (23.3%)
Years post-training			
<1	3 (9.1%)	5 (6.6%)	3 (2.6%)
1–5	10 (30.3%)	6 (7.9%)	17 (14.7%)
6–10	6 (18.2%)	4 (5.3%)	31 (26.7%)
>10	14 (42.4%)	61 (80.3%)	65 (56.0%)

^a^ *UW Psychiatry* University of Washington Psychiatry Residency Program, *HL Psychiatry* Harvard Longwood Psychiatry Residency Program, *UW Medicine* University of Washington Internal Medicine Residency Program, *PGY* postgraduate year

### Ownership themes

We identified ownership themes in five core domains: Physician Actions, Physician Attitudes, Physician Identity, Physician Qualities, and Quality of Patient Care. Themes that emerged in narrative comments from all groups (residents and faculty from psychiatry and medicine programs) are shown in Table 2, together with the number and percentage of respondents whose narrative responses were coded as containing each theme, and representative example quotes for each theme. These themes that emerged in responses from all groups (‘common themes’), as well as themes differing in resident versus faculty and psychiatry versus internal medicine responses, are described below.

**Table 2:** Ownership themes common to residents and faculty members from medicine and psychiatry

Themes	Number (%)	Representative quotes
*Physician actions*		
Advocacy	20 (5.6)	‘… to be their advocate when the clinical course is smooth or rough’
Communication, care coordination	175 (49.2)	‘maintaining communication with other care providers so that continuity will be as smooth and seamless as possible’
Decision making	56 (15.7)	‘taking responsibility for clinical decision making’
Follow through	87 (24.4)	‘I am the one who will follow through and make sure the work on that patient gets done as expected. Things will not fall through the cracks on my watch.’
Knowledge of the patient	51 (14.3)	‘knowing the patient stone-cold’; ‘learning as much as one can about the patient’s condition’
Leadership	12 (3.4)	‘taking a leadership role in the care of one’s patient, whether by being the ‘sole’ person in control, leading a team or appropriately delegating tasks to others’
*Physician attitudes*		
Above and beyond	18 (5.1)	‘going the extra mile’; ‘a commitment to do more the minimum’
‘Buck stops here’	17 (4.8)	‘you are not tagging along behind an attending … you are ‘it’’; ‘I’m responsible for seeing that my patient gets good care and if there are lapses, it’s ultimately on me’
Patient outcome	26 (7.3)	‘feeling invested in whether the patient gets better or not’
Responsibility (feeling)	25 (7.0)	‘to ‘own’ our patients really means, in my view, to feel responsible for their care, to feel the gravity of our interactions, decisions, and actions on their behalf’; ‘Losing sleep if something goes wrong’
*Physician identity*		
Primary care provider	36 (10.1)	‘I am the first person that the nurse and case manager contact … I am also the person who represents the treatment team to the family’
*Physician qualities*		
Initiative	38 (10.7)	‘taking initiative to suggest initial treatments and alterations in treatments where necessary’; ‘Being proactive … rather than assuming someone else has done it’
*Quality of care*		
Best care	13 (3.6)	‘following the golden rule, e. g. am I delivering care that I would want to deliver to a family member or myself’
Comprehensive	30 (8.4)	‘as a physician it means taking ultimate responsibility for every aspect of a patient’s healthcare’
Longitudinal	11 (3.1)	‘the physician … takes the long view … and avoids seeing patient care in terms of a specific, isolated episode’
Patient-centred	29 (8.1)	‘eliciting the patient’s perspective’; ‘trying to help empower the patient in making decisions about their medical care’

### Common themes

Each of the five core domains included at least one theme common to all groups, as follows.

#### Physician actions

Six themes regarding Physician Actions associated with ownership emerged from narrative comments in all groups. These were advocacy for the patient, communication and care coordination, decision-making regarding the treatment plan, follow through in completing patient care tasks, acquiring knowledge about the patient and his/her condition, and leadership of the patient’s care or treatment team (Table 2). The theme communication and care coordination included communication with the patient and family as well as communication with other providers to coordinate care.

#### Physician attitudes

Physician attitudes linked with ownership in all groups were valuing doing more than the minimum required (going ‘above and beyond’), considering oneself to have the final or ultimate responsibility (‘the buck stops here’), and *feeling* responsible both for patient care and for the patient’s clinical outcome and wellbeing (patient outcome).

#### Physician identity

All groups linked ownership with a Physician Identity as the primary or main care provider and the primary contact or ‘go-to’ person for questions regarding the patient and his or her care.

#### Physician qualities

Narratives from all groups identified taking initiative as being integral to ownership.

#### Quality of patient care

Finally, themes of providing the best care for patients, and providing comprehensive, longitudinal, and patient-centred care, were represented in responses from all groups.

### Differences between residents and faculty

While themes in all five core domains were represented across faculty and resident responses, there were differences in the content and emphasis of some of these themes. Within the domain of Physician Actions, residents placed greater emphasis on being the decider and leading the clinical decision-making process, identifying ownership as, for example, ‘*functioning as the primary clinical decision-maker*’, being ‘*the key actor*’, and being the ‘*primary creator of a plan for a patient’s care*.’ One resident posed this issue as ‘*I think the distinction is: does your attending tell you the plan for the patient or do you try to make it yourself, with attending approval?’*

A notable difference between residents and faculty in Physician Attitudes involved the concept of ‘shift’ work. In general, faculty tended to define ownership as having full-time responsibility for one’s patients (‘*I am never really off the clock*,’ ‘*I always in some sense have the patient in mind’, *‘*if needed, stay[ing] overtime to follow through on tasks …, versus handing it off to the next ‘shift*’’). In contrast, residents felt more comfortable sharing patient responsibilities (‘*identifying and designating clear coverage for your patient*’) and commented on the importance of good sign-out. One resident offered that ownership is:

*a dying concept that a single individual physician is responsible for the entirety of non-procedural care that a patient receives. It stems from a period of time when so-called general practitioners followed ‘their patients’ from the outpatient to the inpatient theatre and made care decisions for those patients.*

Regarding Physician Qualities, faculty members discussed the importance of being available to patients (‘*being available to the patient in and outside of regular hours*’, ‘*putting aside administrative/research tasks for the day if the requirements of the individual patient have not yet been met’*).

### Differences between medicine and psychiatry

Both internal medicine and psychiatry responses included themes from all five core domains, with few and minor differences between specialties.

Specifically, within Physician Actions, a theme unique to two psychiatry responses was apologizing for errors (‘*apologize when you’ve made a mistake as your mistake when it is’). *Two themes regarding Physician Attitudes were only abstracted from responses from individual internal medicine residents. These were responsibility extending beyond one’s assigned patients (*‘Being more than peripherally aware of patient that is not specifically assigned to your care’*) and responsibility for the patient care experience* (‘I am responsible for the … patient’s subjective experience while being in the hospital.*’). One medicine resident also suggested that ownership includes a domain of leadership with control or influence as an agent on behalf of the patient (*‘The resident or med student feels … that their actions affect the patient’s care.’*).

## Discussion

In this qualitative study of the definition of ownership of patient care, we have identified a set of core themes common to residents and faculty in medicine and psychiatry across three separate residency programs and two different institutions. These core themes, falling under the overall domains of Physician Actions, Physician Attitudes, Physician Identity, Physician Qualities, and Quality of Patient Care, were advocacy for patients, communication and care coordination, decision-making, follow through, knowledge about the patient, leadership, going ‘above and beyond’, an attitude of ‘the buck stops here’, feeling responsible for patient care and outcomes, identifying as the patient’s primary provider, taking initiative, ensuring the best care, and providing comprehensive, longitudinal, and patient-centred care. Clearly, ownership of patient care is a rich, complex, and multifaceted concept. Despite this, there was substantial agreement across programs and specialties in this study about the meanings of ownership.

To our knowledge, there have been no previous studies of the definition and meanings of ownership of patient care except for our own pilot work [11]. Our current findings agree with the pilot study in identifying core themes of advocacy, communication (including care coordination), follow through, and knowledge of the patient [11]. The remaining core themes from the pilot study—autonomy, commitment, and teamwork—are also represented among common themes in the current study, although the exact descriptive terms used in coding differ. Themes regarding providing the best care and a longitudinal relationship were only noted by faculty in the pilot study, but here were also endorsed by residents, possibly because of the larger sample size. A novel common theme in the current study was the physician’s identity as the primary care provider. Other descriptions of ownership in the literature, although not based on systematic studies of the definition of the term, are varied but each include individual themes identified here [3–10]. Thus, the common themes in our current results appear consistent with and have extended and enriched our prior study and other descriptions of ‘ownership.’

Another aim of the current investigation was to identify differences between residents and faculty members or between specialties. Respondents from Internal Medicine and Psychiatry endorsed a similar variety of meanings of ‘ownership.’ The most striking differences between faculty and residents were seen within Physician Actions and Physician Attitudes, with residents highlighting the importance of decision-making and handoffs, while faculty focused on being a patient’s primary physician for the entirety of his or her care, regardless of being on or off ‘shift.’

Several studies and opinion papers have highlighted the loss of autonomy in current clinical educational settings and have discussed its educational impact [13–17]. Some suggest that increased faculty oversight has led to a decline in resident understanding of ownership [6, 15]. Our results suggest that residents understand and value ownership as much as faculty do, but that changes in clinical education, such as work hour restrictions, shift work, and supervision requirements have led to a change in what ownership means to them. Residents now experience limits in duty hours as the norm and, as such, ownership for them includes being able to provide a quality handoff so that care can continue safely when the resident is not present.

### Educational implications

Our findings have multiple educational implications. Similarities across groups indicate that there is a core, shared understanding of ownership that can serve as the basis for curriculum development. At the same time, the term ownership means many different things to individual respondents. Given the lack of a single ‘consensus’ meaning, educators must be specific in their discussions with residents about what they mean when they use the term.

Many residents in our study valued the role of the decider as part of ownership and prior research suggests that resident autonomy enhances ownership [13]. Providing autonomy balanced with adequate supervision is challenging, especially since resident and faculty assessments of resident abilities may differ [13–17]. Faculty members are more likely to trust and grant autonomy to residents they perceive as competent, confident and motivated [13]. For other residents, clear expectations regarding active engagement in patient care, sensitivity to the resident’s wish for more independence, specific feedback about areas for improvement, and providing carefully graded autonomy may be necessary.

Finally, the largely shared understanding of the meanings of ownership across groups of faculty and residents may provide the basis for discussions of fundamental areas of agreement as well as meaningful discourse about differences. For example, if groups of residents and faculty can recognize that they share basic values, they may be able to engage in discussions about differing perspectives on what it means to display ownership, contrasting traditional views (‘nostalgic professionalism’ [18]) with taking ownership in the context of ‘shift work’. In turn, this may enhance mutual understanding between residents and faculty.

### Strengths and limitations

While qualitative methods do not allow for external validity in the traditional sense of a quantitative generalizability, they do provide the potential for *transferability *of the findings. In this study, transferability was supported through the similarities and differences seen in the themes that emerged across programs, specialties, and institutions. In addition, this study was an expansion of a previous pilot study [11] and our current findings largely replicate and expand those presented in that study. The narrative responses to the structured questions allowed for a level of depth and insight into the research question that could likely not have been achieved with a quantitative approach.

Internal dependability and credibility were achieved by several means. The study was framed and the research questions were asked in a way that assumed a descriptive, qualitative response. We utilized a rigorous analysis plan that included multiple research team coders who engaged in bracketing to minimize *a priori* biases, and who adhered to recognized analytic approaches such as the constant comparative technique [12]. The teams engaged in multiple levels of coding both individually and collectively. The triangulated data from the three programs were developed into codebooks, ensuring that final themes were grounded in data points. The stratification sampling of residents and faculty from multiple disciplines, programs, and institutions successfully achieved data saturation and minimized the opportunity for sample bias.

There are also limitations to the findings from this study. First, because sample adequacy is based on data saturation and not on sampling power, mathematical correlations or causal relationships cannot be inferred. The study was designed to be a qualitative exploration of meanings and experiences related to ownership, identifying component themes. It was not designed to quantitatively assess the relative importance of or prioritize themes. Response frequencies for our common themes cannot be generalized beyond this sample and high frequencies of particular themes related to Physician Actions may reflect our asking respondents to provide examples of behaviours exemplifying ownership. The strength of the large sample size also carried an inherent limitation. Narrative responses to the questions were anonymous and, therefore, it was not possible to return to the individual respondents, to ask for additional insight or clarification. Although themes regarding the meaning of ownership were similar among Internal Medicine and Psychiatry faculty and residents at the two institutions in this study, inclusion of other specialties or sites could yield differing results.

## Conclusion

Our investigation into the definition of ownership resulted in a complex, rich, multivariate understanding of the term. Our study used a qualitative approach to identify elements of this professional concept that emerged from narrative definitions across residents and faculty members from two sites and three programs. Similarities and the few areas of difference between groups can provide the basis for a shared understanding of this term and inform educators on how to best adapt to changing educational demands and learner needs.
